# A Cyber Risk Assessment Approach to Federated Identity Management Framework-Based Digital Healthcare System

**DOI:** 10.3390/s24165282

**Published:** 2024-08-15

**Authors:** Shamsul Huda, Md. Rezaul Islam, Jemal Abawajy, Vinay Naga Vamsi Kottala, Shafiq Ahmad

**Affiliations:** 1 School of IT, Deakin University, Melbourne, VIC 3125, Australia; jemal.abawajy@deakin.edu.au (J.A.); vinaykottala1@gmail.com (V.N.V.K.); 2 BGD e-GOV CIRT, Bangladesh National CERT, ICT Division, Ministry of Post, Telecom and IT, Dhaka 1212, Bangladesh; rezakuet99@gmail.com; 3 Industrial Engineering Department, College of Engineering, King Saud University, Riyadh 11421, Saudi Arabia

**Keywords:** Medical Cyber Physical Systems, Federated Identity Management, cyber risk assessment, OAuth vulnerabilities, SAML

## Abstract

This paper presents a comprehensive and evidence-based cyber-risk assessment approach specifically designed for Medical Cyber Physical Systems (MCPS)- and Internet-of-Medical Devices (IoMT)-based collaborative digital healthcare systems, which leverage Federated Identity Management (FIM) solutions to manage user identities within this complex environment. While these systems offer advantages like easy data collection and improved collaboration, they also introduce new security challenges due to the interconnected nature of devices and data, as well as vulnerabilities within the FIM and the lack of robust security in IoMT devices. To proactively safeguard the digital healthcare system from cyber attacks with potentially life-threatening consequences, a comprehensive and evidence-based cyber-risk assessment is crucial for mitigating these risks. To this end, this paper proposes a novel cyber-risk assessment approach that leverages a three-dimensional attack landscape analysis, encompassing existing IT infrastructure, medical devices, and Federated Identity Management protocols. By considering their interconnected vulnerabilities, the approach recommends tailored security controls to prioritize and mitigate critical risks, ultimately enhancing system resilience. The proposed approach combines established industry standards like Cyber Resilience Review (CRR) asset management and NIST SP 800-30 for a comprehensive assessment. We have validated our approach using threat modeling with attack trees and detailed attack sequence diagrams on a diverse range of IoMT and MCPS devices from various vendors. The resulting evidence-based cyber-risk assessments and corresponding security control recommendations will significantly support healthcare professionals and providers in improving both patient and medical device safety management within the FIM-enabled healthcare ecosystem.

## 1. Introduction

The integration of advanced medical devices and monitoring systems with hospital networks is creating a more connected healthcare environment [1]. This allows for improved collaboration among healthcare providers, potentially leading to higher-quality care at lower costs [1,2,3]. For instance, central monitoring systems like GE’s Clinical Information Central Stations enable real-time observation of patient data collected from wearable devices [4]. Additionally, hospitals can partner with external specialists, expanding their access to expertise and resources [5,6]. To manage user access securely [7] in this complex environment, hospitals are adopting FIM solutions like OAuth, Google AuthSub, and SAML [5,7,8]. These systems ensure authorized personnel, both internal staff and external providers, have access to the information they need, streamlining collaboration and improving patient care.

While these interconnected systems offer significant advantages [1,2,3,9,10,11,12], the increased connectivity creates a larger attack surface, exposing patients’ data to significant cybersecurity risks, including breaches of privacy and data integrity [5,7,8,9,10]. A recent example is the discovery of critical vulnerabilities in GE CICS and Telemetry Servers’ operating system (PwnKit and MDhex) [13,14]. Exploiting these vulnerabilities, attackers could gain unauthorized access (root privilege) to nurses’ stations, potentially compromising patient safety by installing malware, generating false alarms, or tampering with data on connected devices. In the worst-case scenario, data breaches or manipulation could have life-threatening consequences [9,10]. Additionally, FIM solutions within a federated healthcare ecosystem are still under development, and may not be mature enough to address all the security challenges, particularly regarding trust management, data privacy, and efficient identity and attribute exchange [6,15,16,17].

To proactively mitigate these risks, a comprehensive and evidence-based cyber risk assessment is essential. Several studies have explored cyber risk assessment for hospital systems with connected IoMT and medical devices [15,16,17,18,19]. However, these approaches often focus on individual hospitals, neglecting the growing need for interoperable and interconnected healthcare ecosystems. As collaboration among healthcare providers increases, the security challenges shift to a federated framework. This interconnected environment creates a larger attack surface and necessitates a robust risk assessment approach that can identify vulnerabilities and propose mitigation strategies. Existing frameworks [15,16,17,18,19] lack the detail required for such interconnected systems. Compounding these challenges is the limited awareness of cyber threats among healthcare professionals, as evidenced by research showing a third of clinicians struggle to protect patient data during telehealth sessions [12,20]. This highlights the need for improved training and education.

To address this gap, this paper proposes a comprehensive cyber-risk assessment approach specifically designed for collaborative hospitals with connected Medical Cyber Physical Systems (MCPS) [1] and Internet-of-Medical Things (IoMT) [3] devices within an FIM framework. Our novel approach considers a connected hospital environment based on FIM and IoMT protocols across perception layer, network layer, and application layer protocols for attack modeling. This comprehensive analysis allows for the development of more targeted and effective security measures. By identifying and addressing vulnerabilities at different layers of the interconnected system, our framework aims to enhance the overall security posture of collaborative healthcare ecosystems and ultimately reduce the risk of life-threatening situations. The novelty of the proposed approach lies in its focus on the interconnected nature of modern healthcare ecosystems:

- A novel cyber-risk assessment approach has been developed for connected hospitals which considers an FIM framework with connected IoMT and MCPS devices, enabling secure collaboration among healthcare providers to meet next-generation healthcare demands.
- Our approach incorporates a detailed threat modeling methodology for FIM-based federated healthcare ecosystems. This methodology, combining CRR and NIST standards, helps identify, prevent, detect, respond to, and recover from cyber threats across the entire system.
- The proposed framework utilizes a comprehensive, “360-degree” vulnerability assessment approach. This approach considers vulnerabilities in existing IT infrastructure and communication protocols, IoMT and MCPS medical devices, FIM and SSO protocols, including OAuth and SAML, and the combined impact of these vulnerabilities on hospitals.

The proposed risk assessment program and its associated artifacts, which will be presented throughout this paper, serve as crucial tools to address this knowledge gap and empower healthcare professionals to better safeguard patient data. This paper will present these artifacts throughout to address the knowledge gap identified by NIST guidelines (SP 800-50) regarding employee understanding of security vulnerabilities [21]. The rest of the paper is organized as follows. Section 1 presents a comprehensive review of existing approaches to cyber threats in smart hospitals. Section 2 presents the proposed cyber-risk assessment approach, including comprehensive analysis of attacks and their mitigation. Section 3 discusses evaluation of the proposed approach, results of risk analysis and mitigation controls. The last section concludes this research and discusses the future works. Table 1 presents the acronyms and their full meanings which are used throughout this paper.

## 2. Background and Related Work

Vulnerabilities in the IoMT devices combined with network, communication, and authentication protocols cause major data breaches on the health systems [2]. In the USA itself, from August 2020 to July 2021, 706 data breaches were reported, which include 44,369,781 individuals’ private information [22].

For example, Abbott’s ICDs [10] can be compromised through the firmware and communication vulnerabilities, allowing attackers to modify settings and issue commands to drain the batteries of ICDs or inappropriate pacing/shocks. The damages in ICDs will require a surgical procedure to replace batteries and can cause deaths. This allows terrorists to attack the leaders of countries. An example of these vulnerabilities forced US Vice President Dick Cheney to disable wireless features in his implanted pacemaker [16]. Another example is surgical robots [12], which are remotely used in under-developed rural areas, disaster areas, and the battlefield for surgical procedures, allowing smooth and feedback-controlled motions for surgeons via a combination of public and private networks [12]. Prof Alexandre Mottrie of the Department of Urology at the OLV Hospital Aalst, Belgium, commented that robot-assisted surgical procedures can reduce post-operative complications and re-admissions, which significantly reduces overall costs in very busy hospitals with extreme admissions for surgeries [23]. Many hospitals in remote cities often do not have expert surgeons. Surgical robots can be used in these scenarios by third party expert services. However, these robots can be compromised and be taken over for permanent control by the attackers. Then, they can be operated in such a way to cause severe damage to the patients by compromising the software, communication surfaces, installation of backdoors, and eavesdropping [12,20].

In [19], Dong-Won Kim et al. analyzed cyber risk for medical device safety only. They concentrated particularly on the Fennigkoh and Smith model, which considers medical device critical functions, physical risk (PR), and required maintenance only. But they do not consider protocol vulnerability or information security. In [24], M. Kintzlinger and N. Nissim discussed some personal medical devices (PMD), along with their threats, attack flow diagrams, and security mechanisms.

This paper lacks considerations of protocols or overall threats in the healthcare industry. In [25], Mohammed Zaki et al. discussed several attacks on medical devices in smart hospital healthcare systems. Then, they devised a solution using next generation firewall (NGFW) only. In [17], Luigi Coppolino et al. analyzed the top six categories medical devices of eight most important medical assets in smart hospitals, as outlined in the ENISA classification. Among the six categories, the top most category belongs to Diagnostic and Monitoring equipment. The authors devised a SIEM-based monitoring approach here to detect anomalous behaviors of critical medical devices, then correlating them with intelligent feeds and acting accordingly to suggest feasible and appropriate recovery solutions. R. Sreenidhi Ranganayaki et al. [26] reviewed several papers that identify and discuss threats and concerns in healthcare systems. This paper focused on managing the workflow of patient’s data, secure collection, storage, and transmission of clinical patient’s data, and software solutions for the viewing and processing of diagnostic data. The authors also recommended general cyber security solutions, like two-factor authentication, TLS solutions, and multi-layer defense systems to mitigate cyber threats. Gomi et al. [27] introduced an FIM-based delegation model for systems, and proposed a delegation framework that has access control solutions with regards to delegation context. Users can manage their own privileges in the framework, enabling service providers to manage access of entities based on user’s delegated privileges using delegation authority. The authority provides delegation of a delegating entity. This also enables authorities to authenticate a user and manage name identifiers of a user. Kamalanathan Kandasamy et al. [28] studied five dominant cyber-attacks in Asian Hospitals and healthcare institutions, like Trojan, Phishing, Ransomware, Advanced Persistent Threats (APTs), and Malware-Credential Compromises. This paper maps each attack with corresponding National Institute of standards and technology (NIST) cyber risk framework guidelines. Specially, it prescribes some vulnerability self-assessment questionnaires (VSAQ) and risk self-assessment questionnaires (RSAQ) in order to help organization identify their present situation. Zhiqiang Wang et al. [29] studied a medical imaging cyber physical system (MICPS). A medical imaging device threat model and attack vector was discussed in [29], along with a protection mechanism, including data encryption storage, network protection, physical safeguards, system hardening, and security guidance. Remarkably, this paper evaluated 15 medical products, and identified that none of these products fully meet the satisfaction criteria in terms of requirements.

Operationally Critical Threat, Asset, and Vulnerability Evaluation (OCTAVE) Allegro is a tool for risk-based information security strategic assessment and planning. This was developed by Carnegie Mellon University’s Software Engineering Institute. This is suitable for an organization with small teams across business units and IT to address the organization’s security needs [30]. In the literature, there is a risk assessment guideline, called “EBIOS”, available for Information System management. This framework provides general guideline for risk assessment, and lacks more details on different artifacts, such as attack modeling and threat scenarios [31].

Carmel Flash et al. [32] studied most Critical Intensive Care Unit Medical Device (ICUMD) security and ecosystems. The taxonomy of ICUMDs are analyzed, with details including ICUMD security threats, vulnerabilities, and attack building blocks (ABB), which create the attack path and promote actors to penetrate the ICUMD. The 16 cyber threats are mapped with 19 ABBs, and their attack frequency determined. However, this paper was constrained to only ICU and similar medical devices.

Dharmendra Kumar et al. [33] analyzed the critical information infrastructure (CII) risk model. Here, the authors reiterated that advanced technology constitutes managing the security of amalgamated digital, analog, physical, and even human components. The authors emphasized technical-, governance-, and user-level solutions to mitigate cyber threats. The authors mentioned technical-, governance-related, and user-level gaps, and recommended corresponding methodologies and best practices to minimize the gaps. In [34], Z. Zainal Abidin et al. devised a conceptual model of risk assessment for detecting insider threats of cyber physical system (CPS). Then, the authors discussed Monte Carlo and Markov chains for risk assessment model. This model is only a simulation-based model. Logs from servers, like IDS, IPS, or clouds, are collected and analyzed using tools and techniques. The authors developed some insider threat behaviors and analyzed risks using the Monte Carlo model.

In [18], Abouzakhar et al. reviewed threats and attacks on IoMT devices. The authors mentioned that a compromised sensor incorporated into patients’ devices could result in devastating results or could even cost lives. Most IoT devices use IPv6 to fragment big IP packets over a Low Power Personal Area Network (6LoWPAN). This 6LoWPAN is vulnerable to Denial of Service (DoS) attacks. This paper lists significant threats of cloud networks and their countermeasures. Gia et al. [35] presented a 6LoWPAN-based cybersecurity framework for IoMT, which is secure, fault tolerant, and scalable. In this complete architecture, IoMT medical sensor nodes were integrated into 6LoWPAN connects for bio signal acquisition from analog front end (AFE) devices, and were stored in a cloud server for the end users. In [15], Ahmed et al. proposed different metrics to measure the impacts of the cyber vulnerabilities on healthcare IT systems. In [16], Floyd, Travis, and Grieco analyzed the data breach patterns in US hospitals, related vulnerabilities in the hospital systems, and their implications. In [17], Coppolino, L. and D’Antonio et al. proposed a cyber-risk assessment for smart hospitals that considers IoMTs’ system failures, errors related to human when operating IoMTs, and bring-your-own-devices (BYOD) for mobile platforms in conventional IT systems. In [18], Abouzakhar, Nasser, and Jones reviewed the cyber threats in IoMT-enabled hospitals, and proposed a vulnerability assessment approach. In [19], Kim, Dong-Won and Choi et al. developed a method of security assessment of IoMT enabled hospitals to identify and evaluate security threats. Most of the works are focused on a single organization environment, and have limitations in addressing the issues with connected hospital frameworks using FIM-based protocols.

## 3. Proposed Methodology

### 3.1. Proposed Risk Assessment Approach to FIM-Based Hospital Framework with IoMT and MCPS

**Figure 1:**
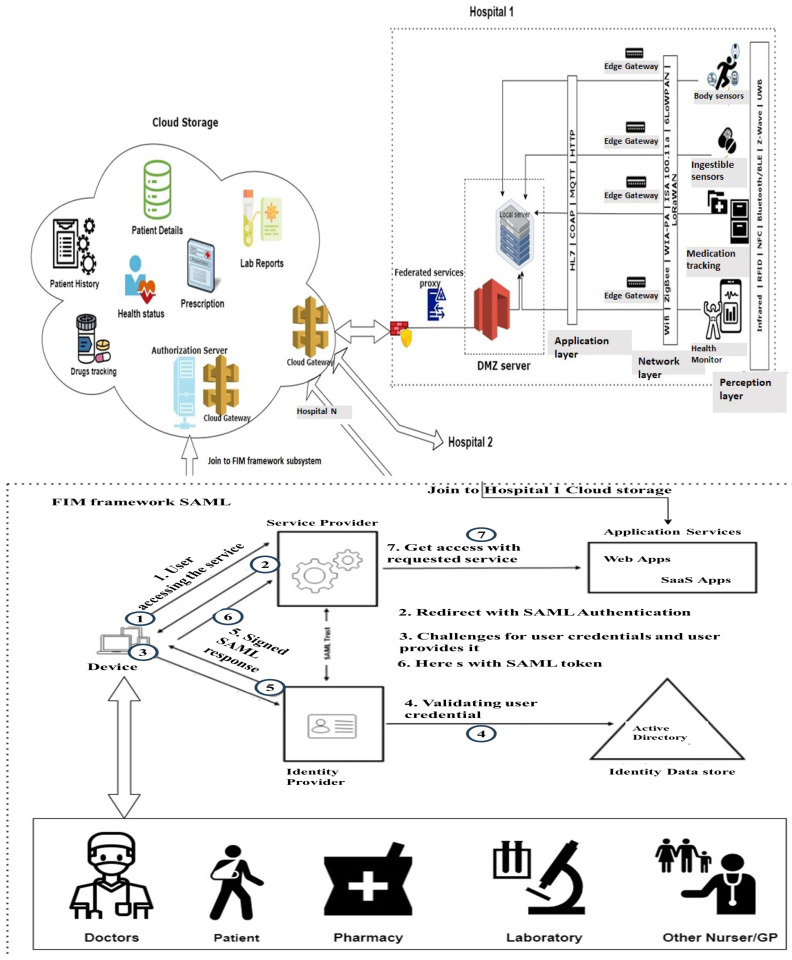
A Federated Identity Management-based hospital framework with connected IOMTs and MCPS.

The proposed approach considers a Federated Identity Management (FIM)-based hospital framework with connected IOMT and MCPS devices. Figure 1 presents the FIM-based hospital framework. Hospital 1: As shown in Figure 1, the top parts of the upper section present the IoMT and MCPS, which are connected to the hospital LAN through the edge gateways, and then edge gateways are connected to the cloud computing network via the DMZ server. The DMZ server and edge gateway sub-networks are properly segmented and segregated by rule-sets to restrict external access to the sensitive IoMT and MCPS devices. On the upper-left side, the hospital network is connected with the cloud network for data storage and running different application services. DMZ protects the hospital’s internal local-area network (LAN) from untrusted traffic. It generally acts as a sub-network between public internet and private networks. It protects the data and filter traffic on the internal networks by providing an extra layer of security using firewalls. The lower section presents how the FIM is implemented. The FIM can use either Security Assertion Mark-up Language (SAML) or OAuth [7].

In the bottom section of Figure 1, service providers and identity providers are together referred to as SAML providers, and are involved in authentication and authorization tasks during the SAML requests. The identity providers (IDP) verify and perform the user authentication. Once the user logs into the identity provider, they will have the access to FIM-enabled applications. Then, IDP and SP work together to authenticate and authorize the users, and grant access to the requested systems/applications. The conversation between the identity provider (IDP) and the service provider (SP) will happen in a message type called SAML assertion, which is an XML document and created by the IDP and verified by the SP. This assertion contains all of the user information that is relevant for that authentication mechanism.

A step-by-step FIM communication process with SAML/OAuth supported protocol is described as below:

1. A clinician would like to access permission for a system/application from a Service Provider (SP); it generates SSOlogin Request, which is sent to the SP.
2. Then, the SP re-sends the SSO login request to the clinician, which then sends the request to the IDP. Here, the clinician’s browser acts as a relay agent.
3. The IDP starts a channel with the clinician for credential verification of client directly, and verifies the client’s identity with the supplied clinician’s username and password.
4. At this stage, there are different versions of the IDP response. In one version, The IDP sends the signed SAML response to clinician which has the authentication status. In other version, the IDP sends a session ID to the SP using the redirect channel, then the SP sends the SSOlogin response to the clinician, which allows the clinician to access the service.
5. Once the SP receives the SAML token/session ID either from the clinician or from the IDP, the SP verifies the authorization privileges.
6. The SP provider grants clinician the access to the requested service.
7. At last, the clinician is able to access to the system/application/service.

The IoMT and MCPS are located in the IoT perception Layer of the IEEE 802.15.4. standard. These devices use protocols like ZigBee, Z-Wave, LowPan, Infrared, RFID, NFC, and Ultra-Wideband (UWB). Many of them are also able to connect using the WiFi protocol. Devices such as ICDs, Automatic Insulin Management Systems (AIMS), surgical robots, pulse oximeters, blood pressure sensors, temperature sensors, portable EKG sensors, and other patient monitoring devices are placed in this layer.

At the network layer, routers, gateways, and access points are located, which use IPV6/IPV4, UIP, SLIP, TCP/UDP, and 6LoWPAN. The most common application layer protocols are COAP, HTTP, MQTT, and HL7, which is used in this framework. HL7 is a set of standards that support exchange, sharing, integration, and retrieval of electronic health information between health entities. It defines the packaging and communication details between various exchange systems. In the upper-left section, cloud storage is the main sub-system. At a device layer, data are collected from different IoMTs, then stored in cloud database through IoT gateway. The cloud gateway in the cloud storage is responsible for transferring the data from the cloud storage to outside users with the help of authorization server of the FIM framework.

### 3.2. Detail Framework of the Proposed Methodology

**Figure 2:**
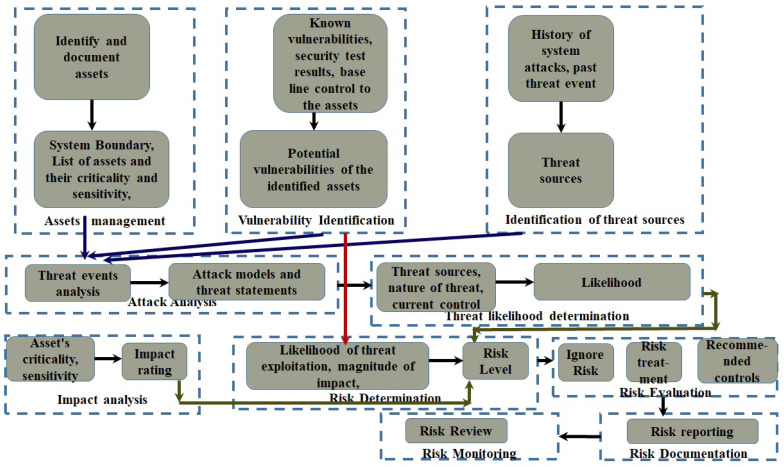
Proposed risk assessment approach to FIM-based hospital framework with IoMT and MCPS.

The proposed cyber-risk assessment approach is based on two frameworks, namely Cyber Resilience Review (CRR) asset management [36] and NIST SP 800-30 [37], to gain advantages from both frameworks. The proposed approach is presented in Figure 2. This involves several stages. In the first part, the asset management from CRR [36] is accomplished. If the organization is running asset management on a regular basis, then the existing asset profile catalog can be used directly. This stage primarily includes identification of different types of assets, such as people, information, technology assets, facility assets, and services. Then, it needs to document all information related to the asset, including the assets’ sensitivity, asset location, asset owners and custodians, services and asset mappings based on dependency, existing controls of assets, services, and the sustained requirements of those [38].

The second step involves identification of the threat sources. The type of threat sources can be (i) hostile cyber or physical attacks; (ii) human errors; (iii) structural failures of organization-controlled resources (e.g., hardware, software, or environmental controls); and (iv) natural and human-made disasters, accidents, and failures beyond the control of the organization. To determine the threat sources, input values can be assumed by the log-history of the systems or threat events (which are events caused by threat sources). The third steps involve identification of vulnerabilities in the existing assets. These can be identified by gathering known vulnerabilities, security test results, and security requirements. By considering these factors, we can determine the potential vulnerabilities of the assets.

The fourth step is to develop the attack models. This will develop attack-model statements/threat events by preparing a deeper analysis, which combines protocol and asset vulnerabilities together, preparing attack trees and attack sequence graphs based on the internal communication of data, user authentications, and data transmissions. The fifth step is to identify the impact of the threat events. The level of impact from a threat event is the magnitude of harm that can be expected to result from the consequences of the unauthorized disclosure of information, unauthorized modification of information, unauthorized destruction of information, or loss of information or information system availability. The inputs here will be the data sensitivity, data criticality, and impact analysis; from these, we will identify the impact rating. The last step is to determine the risk. Risk is a function of the likelihood of a threat event’s occurrence and potential adverse impact should the event occur, which is calculated using Equation (1). After determining the risk, we proceed to risk evaluation. There are four options, as below:

- Accept the risk: if the likelihood and impact rating is low, the risk is accepted.
- Mitigate the risk: in this case, the appropriate security control is applied in place to lower the risk level.
- Transferring risk: risk is transferred to a third party, for example to an insurance company who buys risk as their business [39].
- Risk avoidance: if the risk is extremely high and cannot be mitigated within the current scope of the project, it is avoided.

(1) Risk=VulnerabilityRating∗ImpactRating∗Likelihood

For asset management and the asset catalog, we mainly considered the following categories of assets:

- IoMT and MCPS devices;
- The rest of the IoT ecosystem devices;
- Communication-related devices and infrastructure;
- Platform and backend, application and services, and information.

#### 3.2.1. Threat Modeling in the Proposed Approach for FIM Protocols and IoT Protocols in a Federated Hospital

As mentioned in Figure 1, healthcare staff from different hospitals can access the resources from other hospitals, in which the access relationship is many to many. In an FIM framework, the main protocols are SAML and OAuth. Both of these protocols have a number of vulnerabilities that make the related assets vulnerable. We determine vulnerabilities by attack-sequence diagram analysis.

**Figure 3:**
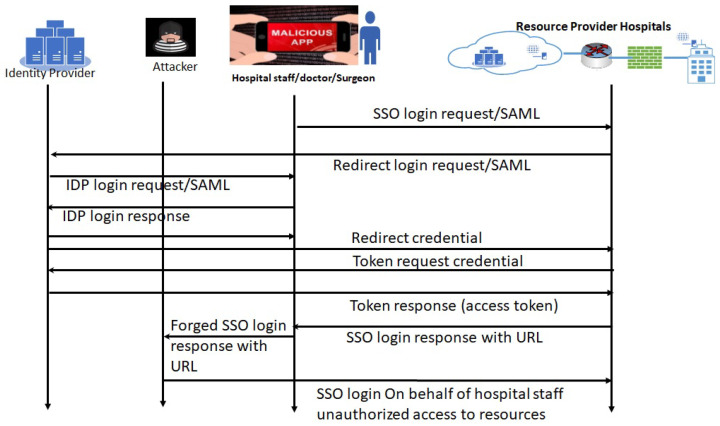
Attack analysis method: an attack sequence diagram when using SAML login into hospital resources.

**1.1 Attack sequence analysis for SAML vulnerabilities in a hospital’s FIM framework:** SAML vulnerabilities can be exploited when hospital staff use their mobile management systems/applications under the FIM framework to access resources in other hospitals/clinics. The clinician logs in to an application using their mobile device, which may already have malicious applications installed. This analysis approach has been explained in the sequence diagram (Figure 3). As we can see in Figure 3, the login credentials or SAML login response with URL are forged by the attacker via the malicious apps in the mobile device. The attacker can use those to access the resources.

**Figure 4:**
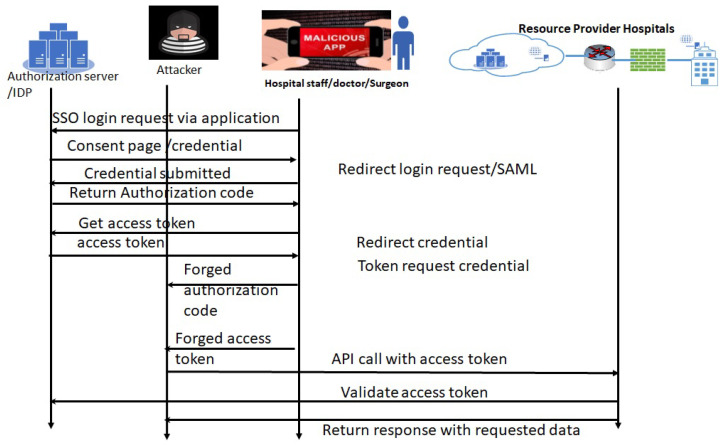
Attack analysis method: an attack sequence diagram when using OAuth-based login into hospital resources.

**1.2 Attack sequence analysis for OAuth vulnerabilities in Hospital FIM framework:** OAuth vulnerabilities can also be exploited, similarly to SAML, when hospital staff use their mobile management systems/applications. This analysis approach has been explained in the sequence diagram (Figure 4). As we can see in Figure 4, OAuth has a different implementation from SAML. OAuth generates the authorization code and access token. Therefore, leaked code or access tokens can be used to access the resources by the attackers. There is no authentication process to verify the authorization server in the OAuth. This vulnerability can also be exploited in the same way as mentioned in the sequence diagram (Figure 4). An attacker can redirect to any other website, and users’ credentials can be stolen. Later, one can be used for logging into the actual authorization server by the attacker.

In the implementation of OAuth in the application, if the access token is sent as a query parameter in the URL, it will be stored in an HTTP “referer”, and can be accessed by other applications and for replay attacks. The OAuth CSRF bug [40] also can help attackers to obtain a valid token, which can be used to access the login for an Outlook e-mail account. A similar CSRF attack can be created in an FIM-based hospital environment.

#### 3.2.2. Threat Modeling in the Proposed Approach for Perception Layer IoT Protocols in a Federated Hospital

**Figure 5:**
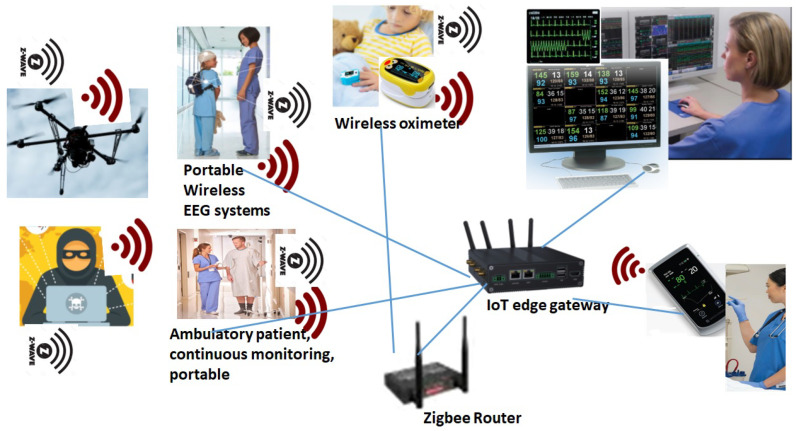
Perception layer devices and their connections to the central nurse station in an FIM hospital framework via Zigbee, Zwave, WiFi, and RF protocols.

IoMT devices, such as portable wireless EEG devices (Nihon Kohden airEEG system), are used for long-term routine EEG monitoring, and also in ICU environments. Patient monitoring devices, such as GZ-120 portable patient monitoring, are used to monitor ambulatory vital signs, including ECG and respiration rate. Several other perception layer devices, which carry ambulatory signals such as ECG, EEG, and heart, include NTX/ZM-540/541PA and ZM-530/531PA Nihon Kohden. The corresponding nurse station central monitoring system, such as Nihon Kohden Defensive Monitoring systems, are connected through a number of IoT protocols, including Zigbee, z-wave, WiFi, and RF, as mentioned in Figure 5.

These IoMT devices can be compromised by the attackers who are in the proximity range of wireless protocols. A recent use of drone technologies can overcome the physical barriers. Therefore, physical barriers cannot stop attackers. Attackers can use different wireless sniffers (e.g., Wireless CC2531 Sniffer Bare Board Packet Protocol Analyzer Module USB Interface Dongle) in the drone, and can come within proximity of the devices. The proposed threat modeling approach develops the following attack sequence diagrams by theoretical analysis of the protocols and device communication behavior. These are later used for the risk determination stage.

**Figure 6:**
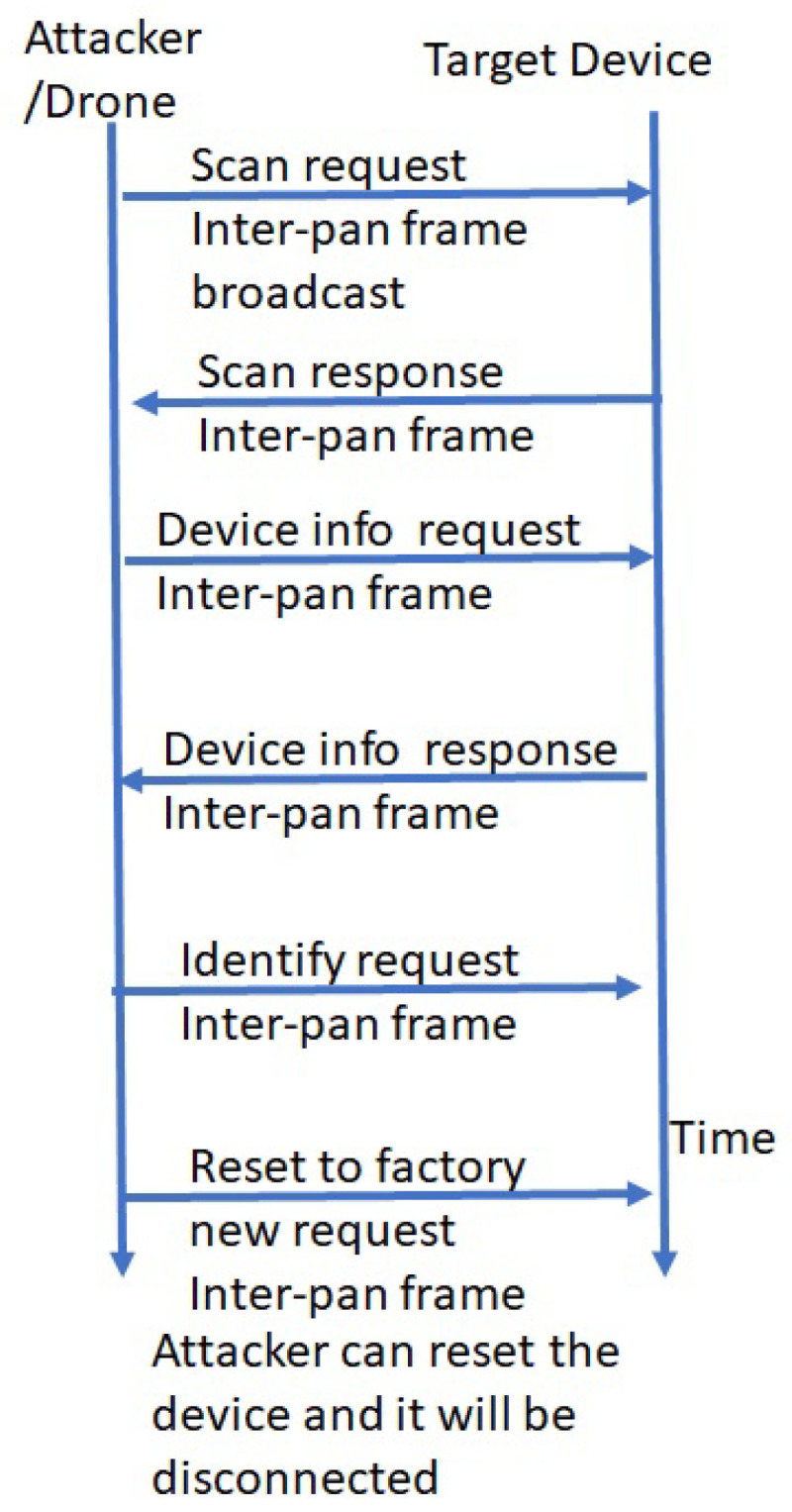
Perception layer devices reset in Zigbee protocol.

**1.1 Attack sequence for IoMT protocols: Zigbee vulnerabilities in FIM Hospital framework:** Figure 6 shows the attack analysis and sequence diagram that can be followed by attackers to disconnect an IoMT device with the Zigbee protocol.

**Figure 7:**
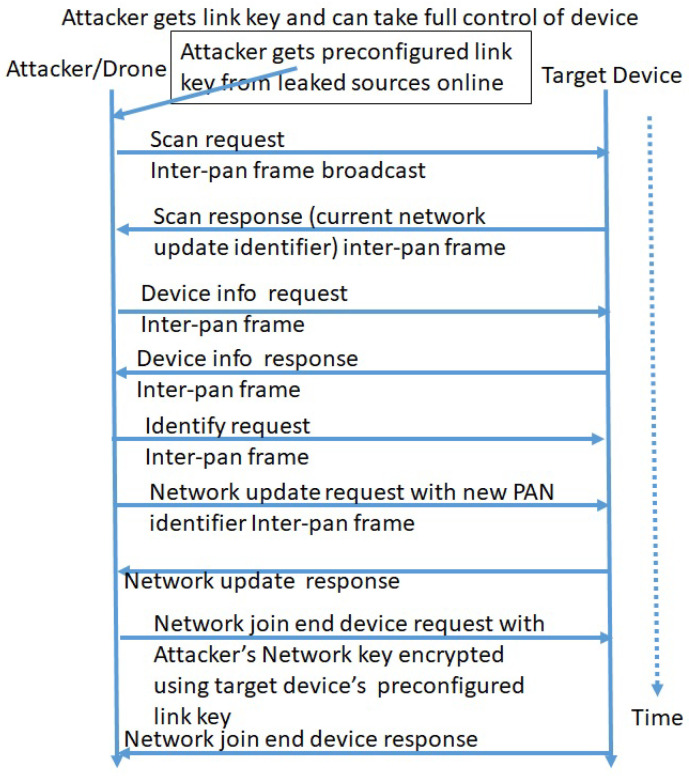
Perception layer devices with Zigbee protocol can be compromised with full control.

The attacker runs a device scan and then sends an identify request through the inter-pan frame. A factory reset command following this will disconnect the device, which can be forced to join in a different (attacker) network. This will allow access to the device’s information and modification of the device settings, which can be later left to join the original network. Then, the device will work as a compromised device in favor of attackers. Figure 7 shows the attack analysis and sequence diagram that can be followed by an attacker to take full control of IoMT devices using Zigbee protocol.

The attacker can use a Zigbee transceiver (Wireless CC2531 Sniffer) to listen the fresh joining conversation (packets). Often, pre-configured link keys are published online. Using the pre-configured link key, the attacker sends a network join end device request, and the attacker’s network key, encrypted using the target device, is a pre-configured link key. Then, the IoMT device will be joined to attacker’s network and fully controlled by attacker. This will allow the attacker to obtain a lot of information from the device. The tampered device can be left to join hospital network again. The same approach can be used to extract the hospital network key and then intercept the data, modify the data, and send it back to the central nurse station. This is more dangerous, and can cause life-threatening situations.

**Figure 8:**
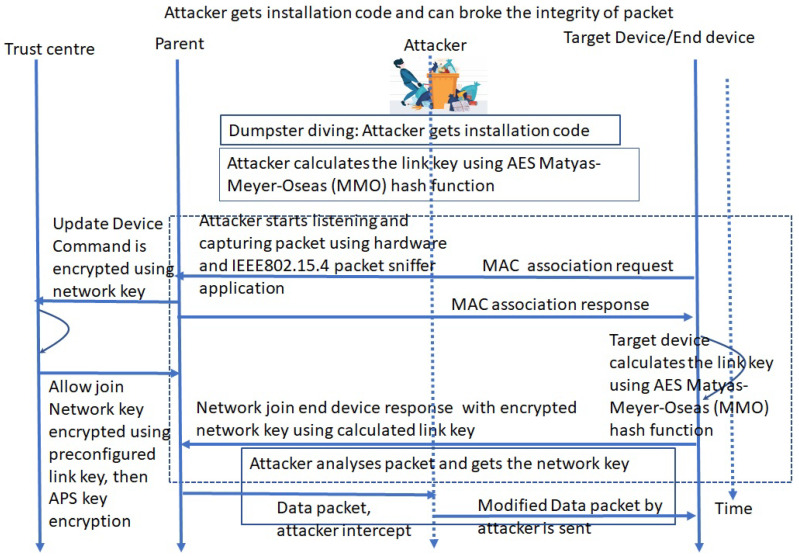
Perception layer devices with Zigbee protocol can be compromised with full control by using installation code.

Instead of a pre-configured link-key, an installation code can be used by an attacker to take full control of IoMT devices. Figure 8 shows the attack analysis and sequence diagram related to the installation code. The attacker gets the installation code through dumpster diving. Then, the attacker calculates the link key using the AES Matyas–Meyer–Oseas (MMO) hash function. The attacker starts listening and capturing packets using hardware and an IEEE802.15.4 packet sniffer. Then, the attacker can send a network join end device response with encrypted network key using calculated link key from installation code. Then, the IoMT device can be taken into attacker network. The attacker can also modify data via the extracted network key of a hospital (the sniffed packet and installation code generated link key), then send modified the data to the central nurse station.

**Figure 9:**
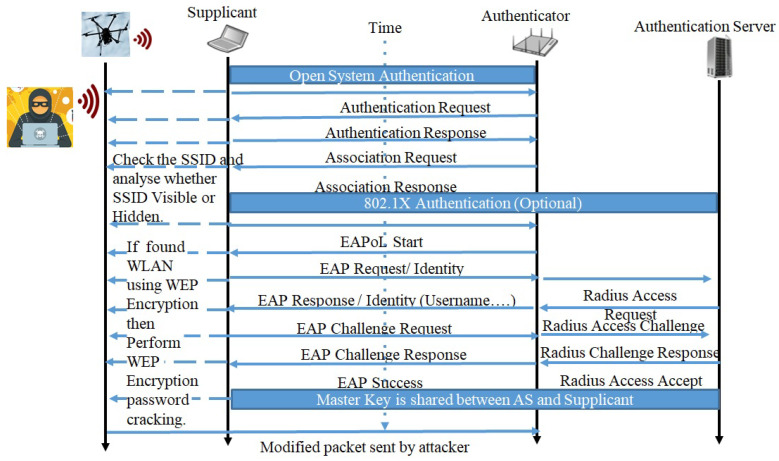
Perception layer devices with WiFi protocol can be compromised by WiFi sniffer.

**1.2 Attack sequence for IoMT protocols: WiFi vulnerabilities in FIM Hospital framework:** Many IoMT devices use the WiFi protocol to connect to the central nurse station or monitoring station. This poses a severe security threat to those devices, due to the known vulnerabilities of WiFi. The attacker continuously scans the SSID and analyzes whether the SSID is visible or hidden, and discovers the IoMT devices that are connected with Wireless Networks. If wireless devices are found in WiFi networks, then the attacker performs password cracking attacks on the protocols WEP, WPA/WPA2, or LEAP Encryption. Currently, a four-way handshake is used in secured WiFi networks. Among these handshakes, the nonce is tricked to reuse the previous key, i.e., reinstalling the already-in-use key. This is called Key Re-installation Attack (KRACK) [41]. In this attack, an attacker forces the involved nonce to reset by identifying and replaying re-transmissions of message 3 of the four-way handshake. As mentioned in the attack sequence in Figure 9, a Pairwise Leader Key (PMK) is generated from association and authentication stage between the Supplicant Nonce (SNonce) and Authenticator Nonce (ANonce). In a message Extensible authentication protocol over LAN (EAPOL) key, ANonce and Unicast messages are sent to the Supplicant. The Supplicant derives the Pairwise Transient Key (PTK).

In the EAPOL key message 2, the Supplicant sends the SNonce a unicast with a Message Integrity Check (MIC). The Authenticator derives the PTK and Group Temporal Key (GTK), if required. The Authenticator sends ANonce, the derived PTK, GTK with MIC to the Supplicant (EAPOL key message 3). In EAPOL key message four, the Supplicant sends the confirmation to the Authenticator. However, an adversary or attacker in the middle intercepts and exploits message 4 and bounds both SNonce and ANonce to send further communication through adversary. Meanwhile, thinking that secure communication already established, the supplicant starts sending message encrypted using PTK and adversary exploits the message. Then, the adversary tricks both ANonce and SNonce to re-negotiate or re-install the previous PTK. Thus, once the PTK is extracted, an adversary or an attacker can modify the data sent by the devices at the perception layer to the nursing station, or vice versa.

## 4. Results and Discussion

**Figure 10:**
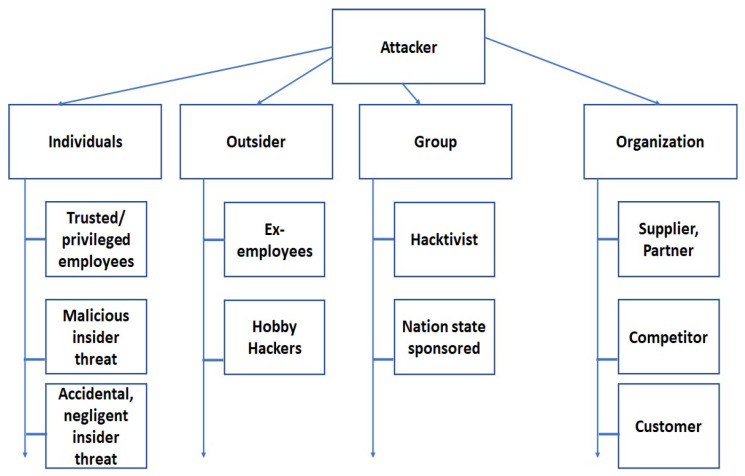
Component of threat model: identified threat sources.

This study includes IoMT and MCPS devices from several companies, including GE healthcare, Ominpod, Nihon Kohden, Abott, Medtronic, and other similar companies. This study considers a large number of devices, including ICDs, blood pressure monitors, EEG, ECG, patient monitoring devices, central monitoring equipment in nurse stations, insulin pumps, telemetry monitors, imaging network-based systems, medical RFID-based inventory systems, and IT systems for healthcare as mentioned in Table 2. We considered both inside and outside threat sources. A list of threat sources is listed in Figure 10. The threat events in the attacks are identified based on the attack analysis explained in the proposed methodology section. Comprehensive attack sequencing has been accomplished, which is then combined and analyzed with the known protocols and device vulnerabilities to identify detailed threat events for attack models.

**Figure 11:**
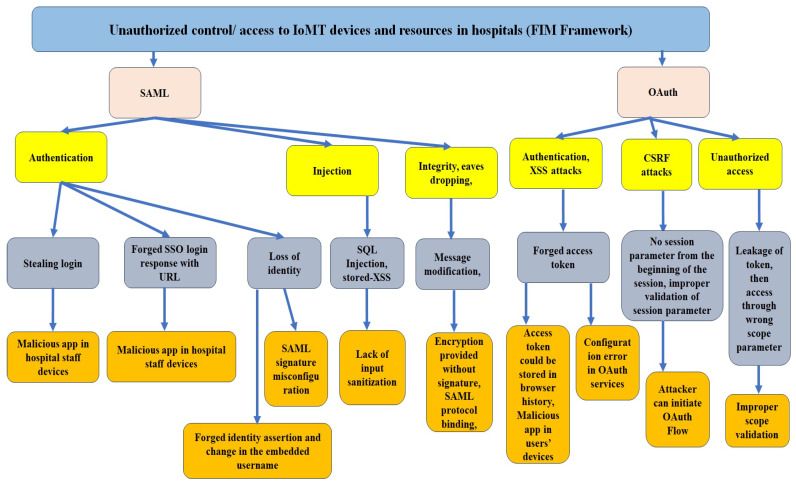
Attack model: attack tree diagram of FIM framework (SAML and OAuth).

**Figure 12:**
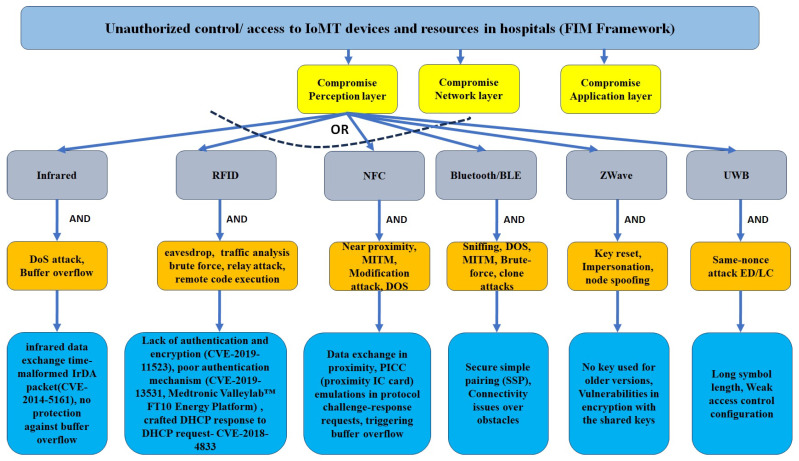
Attack model: attack tree diagram of IoT protocols (left branch).

**Figure 13:**
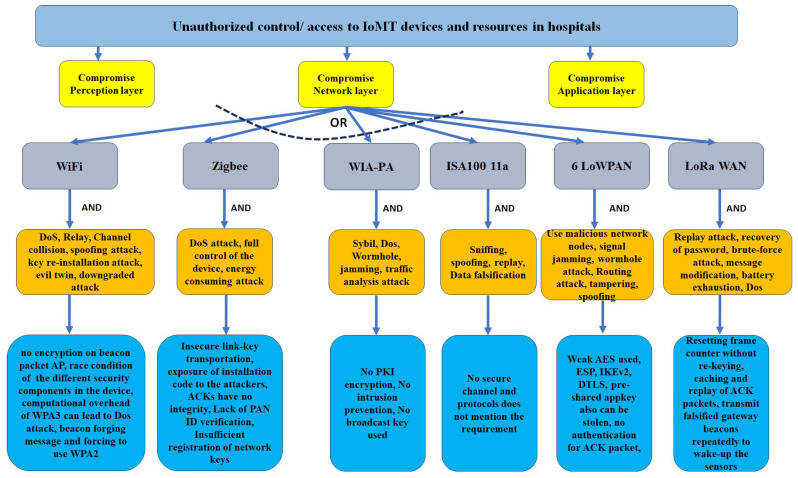
Attack model: attack tree diagram of IoT protocols (middle branch), part of Figure 12.

**Figure 14:**
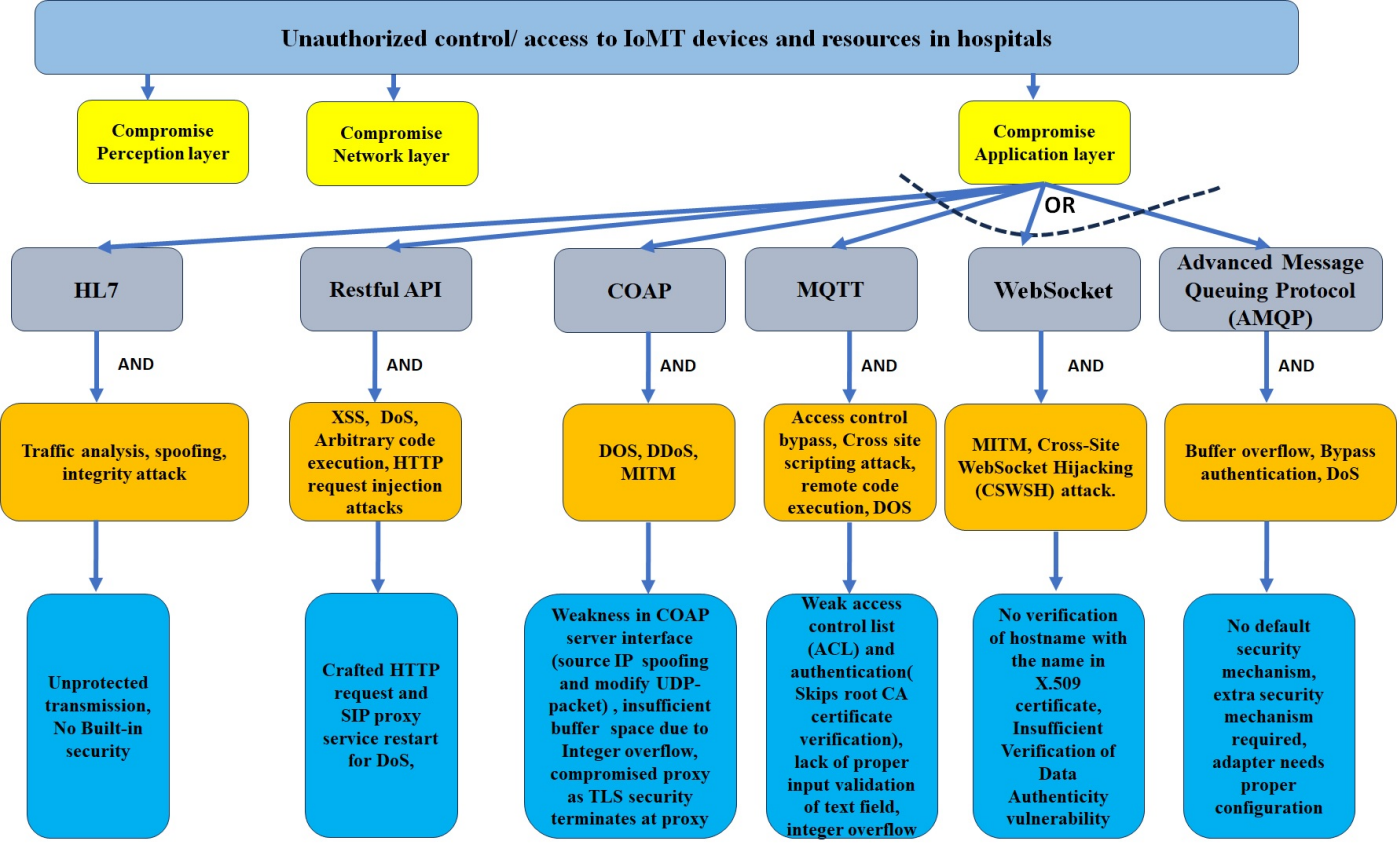
Attack model: attack tree diagram of IoT protocols (right branch), part of Figure 12.

Our detailed threat modeling approach derives the attack tree diagrams mentioned in Figure 11, Figure 12, Figure 13 and Figure 14 for the attack models by using the attack sequence diagrams for FIM and IoT protocols and related devices according to Figure 3, Figure 4, Figure 6, Figure 7, Figure 8 and Figure 9. Each attack is defined by the path of a tree from the root node to a leaf node, as shown in Figure 11. The paths in the tree use the sequences of action, which are defined in our attack sequence diagrams in Figure 3, Figure 4, Figure 6, Figure 7, Figure 8 and Figure 9. An attack uses the event from the sequence diagram, combined with techniques including social engineering, installation of backdoors at users’ devices, brute force attacks, dictionary attacks, and Denial of Service Attacks (DoSyun). The attack models in the threat modeling are the collection of attacks presented by each path in the tree. Then, the attack models are used in the proposed risk evaluation. We calculated cyber-risks for the aforementioned IoMT devices, which are present in Table 3 and also present the corresponding mitigation strategies column. For privacy reasons, we have omitted the names of the companies in Table 3. However, specific vulnerabilities, threat events, attack models, and mitigations are directly applicable to those devices that match corresponding communication protocols and their locations in the FIM framework, as mentioned in the framework (Figure 1 and Figure 5) and in Table 4.

### Risk Determination Treatment and Mitigation

Table 3, Table 4, Table 5 and Table 6 present our final risk calculation for MCPS devices. In Table 3, Table 4, Table 5 and Table 6, all asset groups for IoMT and MCPS infrastructure are considered. Their corresponding weakness and flaws are detailed in these overall IoT asset infrastructure. Then, the corresponding likelihood, vulnerability rating, and level of impact value are assigned to calculate ultimate risk generated from those individual threats for different assets. The risk value indicates the amount of risk for individual medical IoT devices.

## 5. Risk Treatment Control Analysis

**Figure 15:**
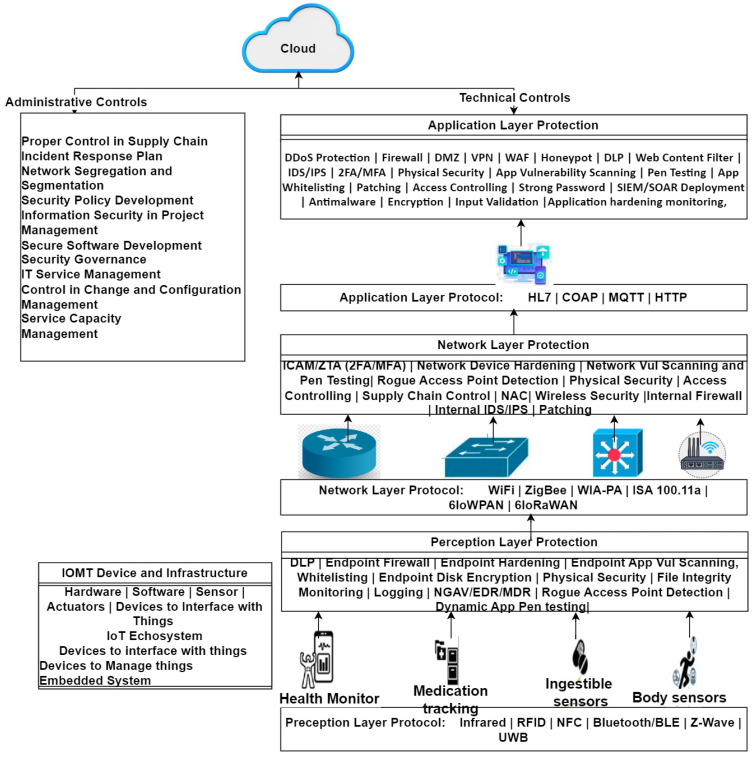
Risk treatment: layer-wise risk treatment control.

The risk treatment columns in Table 3, Table 4, Table 5 and Table 6 present the required security controls for each identified risk. Administrative controls are deployed by establishing security governance. Executive management will be closely engaged in implementing administrative controls. Technical controls for risk treatment are deployed in a layer-wise manner for the perception layer, network layer, and application layer. This will mitigate the risk posed on IoT hardware and software to an acceptable level. In the connected digital healthcare system, both administrative and technical controls are required for a smooth operation (Figure 15).

### 5.1. Administrative Controls

Administrative controls are preventive by nature. These controls are developed and signed by the executive management. They closely lead and monitor the effectiveness of controls. Such controls are: proper control in supply chain, security incident response plan, information security policy development and deployment, information security in project management, secure software development, following the standard guidelines in IT service management, strict controls in change and configuration management, and service capacity management.

### 5.2. Technical Controls

Most technical controls are reactive and detective by nature. For proper risk mitigation in the IoT infrastructure section in Table 3, most technical detective controls are applicable in monitoring and incident response. For IoT devices in perception layer in Table 2 (assets group-1), body sensors, health monitors, medication tracking, ingestible sensors, and actuators, and administrative controls like supply chains, change, and configuration management are required. Technical controls are applicable for identification and mitigation of threat event. Controls like Data Leakage Prevention (DLP), Endpoint Firewall, Endpoint baseline Hardening, Endpoint application vulnerability scanning, Endpoint whitelisting, Endpoint disk encryption, Physical perimeter Security for endpoint devices, File Integrity Monitoring, Logging, Next Generation Antivirus (NGAV)/Endpoint Detection and Response (EDR)/Managed Detection and Response (MDR), Rogue Access Point Detection, and Dynamic App Pen testing are used as primary technical controls, which are mentioned in Table 3 (risk treatment column). To minimize threats posed to networking devices in the network level, such as switches, routers, and gateways, the technical controls for assets group (2,3,4) in Table 2 are to be deployed. The required technical controls for networking devices include Identity, Credential, and Access Management (ICAM)/Zero Trust Architecture (ZTA), Two Factor/Multi Factor Authentication (2FA/MFA), Network Device security hardening, network vulnerability scanning and penetration testing, Rogue Access Point Detection, ensuring Physical Security, Access Controlling mechanisms, Supply Chain Control, Wireless Security, Internal Firewall, Internal Intrusion Detection System (IDS)/Intrusion Prevention System (IPS), and network device patching, as mentioned in Table 3. Supply chain controls also require governance review, site security surveys, and formal security audits as part of the risk mitigation and risk treatment steps.

To mitigate risks in the IoMT Device application software level (assets group 4–5 in Table 2), technical controls are crucial, which are mentioned in Table 4. Technical controls like DDoS Protection, application Firewall, Demilitarized zone (DMZ), VPN, Web Application Firewall (WAF), Honeypot, Data Leakage Prevention (DLP), Web Content Filter, application level IDS/IPS, deploying Two-Factor or multi-factor (2FA/MFA), Physical Security, App Vulnerability Scanning, Pen Testing, App Whitelisting, Patching, Access Controlling, Strong Password policy, Security Incident and Event Management (SIEM)/Security Orchestration and Automated Response (SOAR) deployment, anti-malware, encryption, Input Validation, application hardening, and continuous monitoring are remarkable in preventing attacks on IoMT Devices’ application software.

For threat mitigation in information, network protocol sections for assets (group 6–8 in Table 2), mostly administrative controls are required. For asset groups 6–8 in Table 2, FIM protocols like SAML and OAuth are authentication and authorization statement protocols. These have been explained in Table 5 and Table 6. Even if mutual collusion occurs between any two parties, legitimate entities can use validly generated assertion information maliciously. Thus, a trust policy among involved parties is required, so that parties carry significant liabilities for intentional or inadvertent misuses will address the risk. Again, careful consideration should be taken by issuers and re-players about what to put in the assertion, to store assertion in a remote site, which could be exposed to the attackers. Moreover, SAML is susceptible to identity forge attack, by which user’s identity is exposed to the attackers. This can be prevented by performing blockchain for digital identity management, encryption, digital signing, i.e., TLS encryption and by using reputable SAML, which are the solutions. These are mentioned in Table 5 and Table 6. OAuth risk could be mitigated using blockchain technology and by applying administrative controls. Specially, strong security policy enforcement and vulnerability scanning will reduce the risk in OAuth, which are mentioned in Table 6. At last, classification of assets is the most important parts when applying security controls, which has to be performed appropriately. Classification assigns the appropriate level of protection/security control to the assets based on their values. Some important factors that needs to be considered when classifying assets are: who will have access to the assets and type of access, how long the assets need to be retained, the method used to dispose of the assets, whether data assets require encryption, and appropriate use of assets. It is also important to be aware of the regulations, customers, and business expectations.

## 6. Conclusions

This paper proposes a novel and comprehensive cyber risk assessment approach specifically designed for hospitals utilizing a Federated Identity Management (FIM) framework alongside connected Medical Cyber Physical Systems (MCPSs) and Internet-of-Medical-Things (IoMT) devices. Unlike existing methods, which often have limitations in scope, our approach takes a holistic view, analyzing vulnerabilities across the entire healthcare ecosystem. This includes the ICT infrastructure, potential weaknesses within FIM protocols, and a wide range of IoMT/MCPS devices from various manufacturers (e.g., GE Healthcare, Medtronic). The proposed risk assessment program and its associated artifacts serve as crucial tools to empower healthcare professionals with the knowledge they need to safeguard patient data. This directly addresses the knowledge gap identified by NIST guidelines (SP 800-50) regarding employee understanding of security vulnerabilities. Our initial approach focuses on identifying threats and implementing security controls which constructs our proposed cyber-risk assessment approach for connected hospitals. As the pandemic situation emerged, it is obvious to establish smart healthcare system that can care for and monitor patients more smartly and efficiently. Soon, the smart healthcare system shall require preservation of the privacy of the patients more strictly, according to the related regulations. In future work, we plan to extend this framework to encompass a detailed analysis of privacy risks associated with FIM-based architectures.

## Figures and Tables

**Table 1:** Nomenclature used throughout the paper.

Acronyms	Full Meanings
MCPS	Medical Cyber Physical Systems
IoMT	Internet-of-Medical Devices
FIM	Federated Identity Management
CRR	Cyber Resilience Review
IAM	Identity and Access Management
SAML	Security Assertion Markup Language
KRACK	Key Re-installation Attack
DoS	Denial of Service Attack
WSN	Wireless Sensor Network
DLP	Data Leakage Prevention
CSRF	Cross-Site Request Forgery
ICD	Implantable Cardioverter Defibrillators
OCTAVE	Operationally Critical Threat, Asset, and Vulnerability Evaluation
EDR	Endpoint Detection and Response

**Table 2:** Assets and their vulnerabilities.

Asset Group (ID)	Assets	Vulnerabilities in the Assets
IoMT Device (1)	Hardware	Hardware: design flaw buffer overflow low processing power. Software: 0-day vulnerabilities, firmware or operating system vulnerabilities. Sensor and Actuator: weak encryption or no encryption emanations or radiation from devices by visible or non-visible spectrum that causes data leakage; insecure key storage (availability of link-key online availability of installation code) default link key values; unauthorized commissioning
	Software	
	Sensor	
	Actuators	
Other IoT Ecosystem Devices (2)	Devices to interface with things	Device to Interface: insecure interfaces, improper IT Assets or business processes design inadequate specifications of IT products, design errors. Device to Manage: inadequate usability, policy or procedure flows. Embedded System: lack of mutual authentication between the client and the server.
	Devices to Manage things	
	Embedded System	
Infrastructure (3)	Routers	Routers: buffer overflow, anonymous proxies, vulnerabilities on the routing path. Gateways: lack of appropriate segmentation and security architecture, improper segregation. Power Supply and Security Assets: management protocols are not secure, often no encryption.
	Gateways	
	Power Supply	
	Security Assets	
Platform and Backend (4)	Web-Based Services	Web-Based Services: design flaw, buffer overflow complex monitoring due to high traffic gap between service provider. Cloud Infrastructure: data owner security control mis-configuration inherently exposed to external access.
	Cloud Infrastructure and Services	
Application and Services (5)	Data Analytics and Visualization	Data Analytics: Code injection, SQL injection, Path injection, Inference, aggregation Network and device Management and usages: Default configuration and password, clear text PDU in management protocol, no encryption in management packet exchange
	Devices and Network Management	
	Device Usages	
Information (6)	Patients’ medical record, medical imaging and picture	Lack of proper storage with encryption and poor encryption for data transmission, weak access control.
Authentication: FIM framework (SAML): SAML Protocol, SAML Profiles, SAML artifacts, (7)	Client’s device (BYOD), Organization device, workstation, Clinician’s own workstation and similar devices	SAML binding does not require any authentication, susceptible to all attacks, no authentication and confidentiality requirement for SAML response and assertions, no authentication for service provider to use SAML assertion.
IOT devices: Network protocols, Perception layer, Application layer (8)	Wireless Sensor Network (WSN)	Lack of encryption, SNMP agent default community string, heartbleed bug, factoring RSA export keys, lack of monitoring, insufficient authentication and authorization, poor configuration management, lack of physical security, lack of transport layer encryption, design flaw, buffer overflow, low processing power, default credential, no authentication.
	Radio Frequency Identification (RFID) services	
	Web-Based Services	
	Cloud Infrastructure and Services	
	Data Mining Application	
	Data Processing and Computing	
FIM framework: OAuth (9)	OAuth protocols and related application software	Lack of proper authentication to verify the authorization server, insecure transmission of query parameters in URI, CSRF bug.

**Table 3:** Final risk calculation for a Federated Identity Management Framework-Based Hospital (IoMT Devices and infrastructure).

Assets	Threat Event	Likelihood	Vulnerability Rating	Impact	Risk	Level of Risk	Central Patient Monitoring Devices	IoMT End Devices	MCPS (Surgical Robots, Other MCPS in Hospital)	Risk Treatment
IoMT Device: Hardware	Unauthorized access to facility Theft, Fraud, Sabotage, Vandalism, Hardware malfunction, Supply chain attack	5	3	9	135	Low	low	low	low	1. Applying Physical Control; 2. Proper Control in Supply Chain.
IoMT Device: Software	Access to device software, Alteration of Software, Abuse of 0-day Vulnerabilities	8	7	9	504	High	High	High	High	1. Manage and maintain strong password policy; 2. Update on patch and technology; 3. Regular Vulnerability Assessment and Penetration Testing (VAPT); 4. Disable unwanted functions; 5. Anti-DDoS tool; 6. IPS, IDS installation; 7. Monitoring and Update.
IoMT Device: Sensor	Rating modification, Deletion Supply Chain attack	8	4	8	256	Moderate	Moderate	Moderate	Moderate	1. Proper Monitoring; 2. Proper Control in Supply Chain.
IoMT Device: Actuators	Loss of integrity via compromised communication	5	7	7	245	Moderate	Moderate	Moderate	Moderate	1. Proper Monitoring; 2. Proper Control in Supply Chain.
IoT Ecosystem: Devices to interface with things	Supply Chain attack	5	3	5	75	Very Low	Very Low	Very Low	Very Low	1. Message encryption; 2. Anti-DDoS, Backup; 3. Proper Control in Supply Chain.
IoT Ecosystem: Devices to Manage things	“Man in the middle Supply Chain attack”	8	3	5	120	Low	Low	Low	Low	1. Message encryption; 2. Anti DDoS; 3. Backup; 4. Proper Control in Supply Chain.
IoT Ecosystem: Embedded System	“Eavesdrop attack, Spoofing attacks. Replay attack, Message Deletion Modification, DOS attack”	5	5	9	225	Moderate	Moderate	Moderate	Moderate	1. Proper Control in Supply Chain; 2. Message encryption.
Infrastructure: Routers	Sniffing, DoS, MITM, Brute-Force, device duplication attacks.	9	7	9	567	High	High	High	High	1. Anti-DDoS tool; 2. IPS installation; 3. Monitoring and Update; 4. Network segmentation and segregation; 5. Appropriate boundary protection; 6. Installation of DMZ server; 7. separate critical data assets and critical devices.
Infrastructure: Gateways	Key Reset, impersonation, node spoofing, Black Hole attacks.	9	7	9	567	High	High	High	High	Monitoring and Incident response
Infrastructure: Power Supply	ED/LC, Same-Nonce attack.	9	7	8	504	High	High	High	High	1. Monitoring and Incident response; 2. Physical Security Control.
Infrastructure: Security Assets	Web application attacks, injection attacks (Code injection: SQL, XSS) DoS, DDoS, Replay, Channel collision, Application layer attack, i.e., Ping of Death, XDoS, WinNuke, HTTP Floods	9	9	9	729	Very High	Very High	Very High	Very High	1. Anti-DDoS tool; 2. IPS/IDS installation; 3. Monitoring and Update; 4. EDR, XDR, MDR deployment; 5. ICAM/ZTA (2FA/MFA); 6. Device Hardening; 7. Vulnerability Assessment and Penetration Testing; 8. Patching.

**Table 4:** Final risk calculation for Federated Identity Management Framework-based Hospital (Platform and Backend).

Assets	Threat Event	Likelihood	Vulnerability Rating	Impact	Risk	Level of Risk	Central Patient Monitoring Devices	IoMT End Devices	MCPS (Surgical Robots, Other MCPS in Hospital)	Risk Treatment
Platform and Backend: Web-Based Services	Installing default link keys or sending security headers in clear text on auxiliary frames, logging that causes DoS, uses of Initiation Vectors which may lead to key compromise, energy-consuming attacks.	9	9	9	729	Very High	Very High	Very High	Very High	1. Anti-DDoS tool; 2. IPS installation; 3. Monitoring and Update; 4. Strong remote access mechanism; 5. Logging and alert system to targeted server functions; 6. Regular scanning and evaluation of interface and code, including proper control for configuration and change management.
Platform and Backend: Cloud Infrastructure and Services	Sybil, DoS, wormhole, Jaming, traffic analysis attack.	5	5	9	225	Moderate	Moderate	Moderate	Moderate	1. IPS installation; 2. Anti-Virus, Malware deployment; 3. Monitoring and Update; 4. Robust configuration/change control; 5. Data storage communication encryption; 6. Training of system administrators.
Application: Data Analytics and Visualization	Replay attacks, recovery of passwords, malicious message modification, battery exhaustion and DoS.	9	5	9	405	Moderate	Moderate	Moderate	Moderate	1. Message encryption; 2. Anti-DDoS deployment; 3. Input validation, output throttling, Anonymization; 4. SIEM/SOAR deployment; 5. Application Hardening, Monitoring; 6. WAF, Honeypot, DLP; 7. Encryption; 8. Web Content Filtering.
Application: Devices and Network Management	Spoofing or integrity attacks, Flooding attacks, Worms/Trojans, Rootkits, Elevation of Privileges,	9	5	9	405	Moderate	Moderate	Moderate	Moderate	
Application: Device Usages	Eavesdropping- theft- breach and manipulation, flooding attacks, Abuse of Information Leakage	9	9	9	729	Very High	Very High	Very High	Very High	
Patients’ medical record, medical imaging and picture	At Rest: Parsing, Cache, amplification, spoofing. Cross-protocol attacks.	9	9	9	729	Very High	Moderate	low	low	1. Boundary protection; 2. Strong password; 3. Encryption in storage; 4. Antimalware; 5. Port authentication; 6. Log monitoring; 7. SIEM Deployment; 8. Output throttling system; 9. Tokenization of data; 10. Regular backup; 11. Data Loss Prevention (DLP).
Patients’ medical record, medical imaging and picture	In Transit: Traffic analysis, Port Obscurity, sniffing and password cracking in wireless communication, obtain user detail, installation of backdoor on digital imaging and communications in medicine (DICOM) server	9	9	9	729	Very High	Moderate	low	low	
Patients’ medical record, medical imaging and picture	In use: Import of patient data from media storage which has malware embedded, back door installation in this way	8	8	8	512	High	Moderate	low	low	

**Table 5:** Final Risk Calculation for Federated Identity Management Framework-based Hospital (FIM and SAML).

Assets	Threat Event	Likelihood	Vulnerability Rating	Impact	Risk	Level of Risk	Central Patient Monitoring Devices	IoMT End Devices	MCPS (Surgical Robots, Other MCPS in Hospital)	Risk Treatment
FIM SAML: Cleint’s device (BYOD), Organization device, workstation,	Eavesdropping: -Theft of user authentication info -Theft of bearer token, malicious app installed in client’s device, XMLDSIG’s canonicalization algorithms provides weak protection that allow attackers to bypass authentication by creating identical cryptographic signature using XML documents, XML parsing problem can cause incorrect authentication in the SAML assertion	9	9	9	729	Very High	Very High	Low	Very High	1. Educate clinicians; 2. Monitoring log alert system; 3. Tracking clients; 4. Proper Installation of SAML scanner: SOAP encryption and message integrity; 5. SOAP binding level digital signature; 6. Authentication for service provider for SAML assertion and artifacts match; 7. Change management of servers; 8. Regular evaluate and asses SAML codes; 9. Mobile device management installed; 10. Limit apps installation; 11. Blockchain for digital identity management; 12. Managing privacy preferences.
FIM SAML: Cleint’s device (BYOD), Organization device, workstation.	Replay	2	5	5	50	High	Very High	Low	Very High	
FIM SAML: Cleint’s device (BYOD), Organization device, workstation,	Message Insertion	2	5	5	50	Very Low	Very High	Low	Very High	
FIM SAML: Cleint’s device (BYOD), Organization device, workstation,	Message Deletion	2	5	5	50	Very Low	Very High	Low	Very High	
FIM SAML: Cleint’s device (BYOD), Organization device, workstation,	Message modification	8	8	8	512	High	Very High	Low	Very High	Continue risk treatment from FIM SAML: Cleint’s device (BYOD), Organization device, workstation,
FIM SAML: Cleint’s device (BYOD), Organization device, workstation,	Man in the middle	9	8	10	720	High	Very High	Low	Very High	
IOT devices: Wireless Sensor Network (WSN)	Jamming, Tampering, Exhaustion, Collision, Unfairness	8	8	10	640	High	High	High	High	1. Frequency Hopping Spread Spectrum (FHSS); 2. Direct Sequence Spread Spectrum (DSSS); 3. Regulated transmitted power; 4. Raising Alarm; 5. Rate Limiting; 6. Error-Correction Code; 7. Small Frames Transmission.
IOT devices:Radio Frequency Identification (RFID)	Permanently disable tag, Temporarily disable tag, Replay Attack	7	8	10	560	High	High	High	High	1. Public-key cryptography; 2. Distance Limiting.
IOT devices:Web-Based Services	DoS, Replay, Channel collision, Spoofing attacks.	5	5	10	250	Moderate	Moderate	Moderate	Moderate	1. Public-key cryptography; 2. Strong Password Management; 3. Monitoring; 4. Supply chain Monitoring; 5. Anti-malware tool; 6. Port authentication; 7. Network and Application policy enforcement, etc.
IOT devices: Cloud Infrastructure and Services	Installing default link keys or sending security headers in clear text on auxiliary frames, Energy-consuming attacks.	5	5	8	200	Moderate	Moderate	Moderate	Moderate	
IOT devices: Data Mining Application	Sybil, DoS, Wormhole, Jaming, Traffic analysis attack.	7	7	7	343	Moderate	Moderate	Moderate	Moderate	
IOT devices: Data Processing and Computing	Use of malicious intermediary network nodes, Signal jamming, traffic analysis, attackers selectively prevent correct packet reassembly.	10	8	10	800	Very High	Very High	Very High	Very High	Cont. same risk treatment of IoT devices as before.

**Table 6:** Final Risk Calculation for Federated Identity Management Framework-based Hospital (OAuth-related services and applications).

Assets	Threat Event	Likelihood	Vulnerability Rating	Impact	Risk	Level of Risk	Central Patient Monitoring Devices	IoMT End Devices	MCPS (Surgical Robots, Other MCPS in Hospital)	Risk Treatment
OAuth-related services and applications	ARP Spoofing, Cache Poisoning, Cross-Site scripting, Man-in-The-Middle (MITM), Drown attack, Denial of Service(DoS) attack, buffer overflow, injection attack, hijacking, Flooding Attacks, EternalBlue attack, Trojan horse attacks, shell script attacks, directory harvest attack, Malfunction, Remote Code Execution, Unauthorized Access.	9	9	9	729	Very High	Very High	Very High	Very High	1. Educate clinicians; 2. Monitoring log and use of alert system; 3. Tracking of clients location; 4. Patching updating of systems; 5. Configuration and change management of systems; 6. Regular evaluate and asses OAuth interfaces and codes; 7. Mobile device management software installation including device tracking, wiping, policy enforcement; 8. Limit apps installation; 9. Use tools to verify between code and user, in the implementation redirect-URL should use SSL TLS; 10. Use of vulnerability scanner such as OAuth scanner; 11. Perform Blockchain for digital identity management (consider managing privacy preferences) in healthcare system; 12. Perform industrial IoT Blockchain to secure searchable encryption approach.
OAuth-related services and applications	Replay attack	7	7	7	343	Moderate	Very High	Moderate	Very High	
OAuth-related services and applications	Message modification	5	5	5	125	Low	Very High	Moderate	Very High	
OAuth-related services and applications	Man in the middle, stolen credential	9	9	9	729	Very High	Very High	Moderate	Very High	Cont. same risk treatment from OAuth-related services and applications
OAuth-related services and applications	Impersonation attack: Stolen access token, stolen authorization code, malicious app installed, mis-configuration and incorrect implementation of interface in the web application for implicit grant flow and authorization code allows cross-site request forgery (CSRF) and redirection of endpoint to the attackers.	9	9	9	729	Very High	Very High	Moderate	Very High	1. Educate clinicians; 2. Monitor the log and use of alert system, 3. tracking of clients location; 4. Patching updating of systems; 5. Configuration and change management of servers; 6. Regular evaluate and asses OAuth interfaces and codes; 7. Mobile device management software installation including device tracking, wiping, policy enforcement; 8. Limit apps installation; 9. Use tools to verify between code and user, in the implementation redirect-URL should use SSL/TLS, 10. Use of vulnerability scanner such as OAuth scanner; 11. Perform industrial IoT Blockchain to secure searchable encryption approach.

## Data Availability

Data are contained within the article.
